# Masa ciała dzieci w wieku szkolnym i powiązanie jej z produktem krajowym brutto

**DOI:** 10.34763/devperiodmed.20172103.179185

**Published:** 2017-10-28

**Authors:** Aneta Grajda, Zbigniew Kułaga, Beata Gurzkowska, Magdalena Góźdź, Małgorzata Wojtyło, Mieczysław Litwin

**Affiliations:** 1 Public Health Department of the Children’s Memorial Health Institute, Warsaw, Poland; 2 Nephrology and Hypertension Department of the Children’s Memorial Health Institute, Warsaw, Poland

**Keywords:** GDP per capita, obesity, overweight, school-age children, underweight, PKB per capita, otyłość, nadwaga, dzieci w wieku szkolnym, niedowaga

## Abstract

**The aim:**

To examine the possible relationship between the prevalence of underweight, overweight and obesity of Polish children and adolescents and the gross domestic product per capita (GDP).

**Material and methods:**

This report is based on the results of a nationwide, representative population study. An area of the country, the so-called Eastern Poland, where GDP per capita is less than 80% of the national average, was designated, based on the data from the Central Statistical Office of Poland (CSOP) and was compared with the rest of the country. The frequency of underweight, normal weight, overweight and obesity, as well as the odds ratio were calculated for gender and region of residence. The body mass index (BMI) for age and sex was standardized and expressed as z-score. The statistical significance of standardized mean differences of BMI depending on the area was examined using the t-test.

**Results:**

There were significant differences in the prevalence of underweight, overweight and obesity between the regions analyzed. The region of Eastern Poland was characterized by a lower risk of overweight and obesity and an increased risk of underweight in comparison with the rest of the country.

**Conclusions:**

Identification of the diverse prevalence of overweight and underweight depending on the region and determined according to the economic status is an opportunity to modify and adapt the strategy of implementing programs aimed at promoting healthy behaviours.

## Streszczenie

**Cel:**

Zbadanie związku między niedowagą, nadwagą i otyłością dzieci i młodzieży a produktem krajowym brutto (PKB) przypadającym na 1 mieszkańca.

**Materiał i metody:**

Praca opiera się na wynikach ogólnopolskiego, reprezentacyjnego badania populacyjnego. Obszar kraju nazwany Polska Wschodnia, gdzie PKB na 1 mieszkańca jest poniżej 80% średniej krajowej, został wyznaczony na podstawie danych Głównego Urzędu Statystycznego (GUS) i był porównywany z resztą kraju. Wyznaczono częstość występowania niedowagi, masy ciała w normie, nadwagi i otyłości oraz iloraz szans (IS) w zależności od płci, regionu zamieszkania. Wskaźnik masy ciała (BMI) dla wieku i płci standaryzowano i wyrażono w postaci z-score. Istotność statystyczną różnic średnich standaryzowanych BMI, w zależności od regionu zamieszkania, badano testem t.

**Wyniki:**

Wykazano istotne różnice w występowaniu niedowagi, nadwagi i otyłości między analizowanymi regionami. Obszar Polski Wschodniej charakteryzował się mniejszym ryzykiem wystąpienia nadwagi i otyłości, natomiast większym ryzykiem wystąpienia niedowagi w porównaniu z resztą kraju.

**Wnioski:**

Identyfikacja zróżnicowania częstości występowania nadwagi i niedowagi w zależności od regionu wyznaczonego na podstawie statusu ekonomicznego staje się okazją do modyfikacji i dopasowywania strategii działań w zakresie realizacji programów mających na celu promocję zachowań prozdrowotnych.

## Introduction

The observed steady increase in the prevalence of overweight and obesity affects both developed and developing countries [1]. Socio-economic, cultural and lifestyle differences influence the incidence of overweight and obesity, as well as underweight, especially at the developmental period of life [2]. In Poland the prevalence of overweight (including obesity) was noted as higher compared to the prevalence of underweight in the majority of provinces [3]. Moreover, differences in the nutritional status between pupils in urban and rural areas were reported [4]. differences in the prevalence of overweight (including obesity) between regions of the country may reflect the diversity of risk factors for obesity at the individual or area level (or both) [5]. Economic development and the socioeconomic situation impact the changes in diet in areas with both high and low income [6], and thus the nutritional status of the population in the developmental period [7, 8]. Gross domestic product (GDP) per capita is a basic measure of prosperity in economic and social sciences. Regional differences of GDP per capita may be related to the diversity of the nutritional status. GDP per capita in Poland has for years been showing large regional differences. In five provinces − located in the Eastern area of the country – the GDP per capita is less than 80% of the national average. This level puts these provinces at the end of the ranking of regions with the lowest GDP per capita in the European Union (less than 40% of the average in the EU) [9]. Knowledge of regional differences in the health status of the developmental period population may be useful in diagnosing the obesogenic environment and then taking appropriate actions aimed at reducing the prevalence of overweight and obesity, which is currently among the priorities of the activities financed by public funds [10].

## The aim of the study

The aim of this study was a comparison of the prevalence of underweight, overweight and obesity in children and adolescents in Poland depending on the region of residence determined on the basis of GDP per capita.

## Material and methods

Data from the representative study “Elaboration of the reference range of arterial blood pressure for the population of children and adolescents in Poland” − PL0080 OLAF, conducted by a team of researchers from the Children’s Memorial Health Institute (CMHI) in Warsaw in cooperation with researchers from around the country were used for this analysis. The survey was conducted after the approval of the Bioethics Committee at CMHI. The study population consisted of pupils aged 6-19 years old attending primary schools, lower secondary schools and upper secondary schools across the country in the years 2007-2009. A two-stage sampling was performed; the primary unit of sampling was school. The basis for creating a sampling frame were the lists of all schools in Poland obtained from the Ministry of Education. Before the sampling, schools were stratified. With regard to primary and lower secondary schools, urban and rural areas constituted the strata, while various types of schools were the strata in the case of upper secondary schools. Sampling the schools, with probability proportional to the size of the unit, was conducted separately for each stratum. In the second phase, based on the numbers of students in the school, sampling without replacement was carried out. The survey was conducted after obtaining written consent from the parents of the pupils who were under 18 years old and from students who were over 16 years old. The children’s calendar age was calculated based on the difference between the date of birth and the date of the examination. The results were entered in the decimal system and the calendar age was determined as the mid-range (e.g. children within the range of ≥6.5 and <7.5 years are 7 years old). Measurements of height and weight were carried out according to the protocol of the OLAF study [11, 12]. On the basis of the measurements, arithmetic means of height and weight were calculated. BMI was calculated from the following formula:

BMI = body weight (kg)/[height(m)]^2^.

The factual basis for grouping by region was the GDP level. Provinces of GDP below 80% of the national average are located in the eastern parts of the country, and therefore this region is called Eastern Poland for the purpose of this publication. Eastern Poland included the following provinces: lubelskie, podkarpackie, świętokrzyskie, warmińsko-mazurskie and podlaskie. For the purpose of this publication the other provinces were included in the area called “the rest of the country”. The data were grouped by sex and one-year age categories in the regions of residence of the study’s participants, i.e. the Eastern Poland region and the rest of the country. The results were analyzed using the SAS 9.2, EpiInfo 3.5.1. and LMSgrowth (downloaded from: http://homepage.mac.com/tjcole/FileSharing1.html) software packages. Body weight status was categorized as overweight and obesity according to BMI values, based on the International Obesity Task Force (IOTF) recommended age- and sex-specific cut-off criteria [13], and into underweight according to BMI cut-off points proposed by Cole TJ et al [14]. Body weight categories were assigned with the use of LMSgrowth software - a Microsoft Excel add-in. The frequency of underweight, normal weight, overweight and obesity, and the odds ratio (OR) with 95% con$dence intervals (CI) were calculated for children and adolescents aged 6-19 years, depending on gender and region of residence. BMI was standardized relative to the Polish 2010 Growth references and expressed as the z-score [12]. The statistical significance of differences in the frequency of underweight, normal weight, overweight and obesity between the regions were assessed using the Chi-square test for the whole sample and separately by gender. The statistical significance of standardized mean differences of body mass index (BMI) depending on the area was studied using the t-test. The statistical significance was assumed at p <0.05.

## Results

In the period between November 2007 and November 2009, 17 573 pupils aged 6-19 years were examined. The analysis of the frequency of underweight, overweight and obesity was carried out on data from 17 550 participants (including 8386 boys − 47.8%, 9164 girls − 52.2%) excluding data from 16 pupils due to the incompleteness of height and/or weight measurements and data from seven girls who were pregnant. Data from 4,164 inhabitants of Eastern Poland and 13,386 inhabitants of the rest of the country were analyzed. In the analysis of standardized mean differences of body mass index (BMI), depending on the region of residence, data from 17,427 pupils aged 6.5-18.5 years (including 8327 boys and 9100 girls) were considered, and the data from 47 participants below the age of 6.5 years and 76 over the age of 18.5 years were excluded.

### Region of residence − Eastern Poland in comparison to the rest of the country

**Fig. 1:**
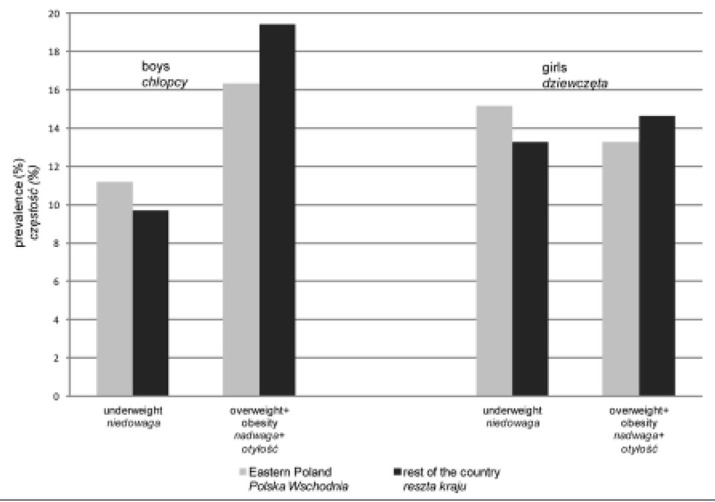
Prevalence of underweight, overweight and obesity among boys and girls aged 6-19 years by region of residence. Ryc. 1. Częstość występowania niedowagi, nadwagi i otyłości wśród chłopców i dziewcząt w wieku 6-19 lat według regionu zamieszkania.

Statistically significant differences were proven in the frequency of underweight, overweight and obesity among children and adolescents between the Eastern Poland region and the rest of the country in the whole study sample, as well as among boys with overweight (including obesity) and girls with underweight (tab. I) (fig. 1). The Eastern Poland region with GDP per capita below 80% of the national average was characterized by a lower risk of overweight and obesity and an increased risk of underweight in comparison with the rest of the country.

**Table I:** Prevalence (%) of underweight, normal weight, overweight and obesity among 6-19 year-old children and adolescents depending on gender and region of residence (Eastern Poland vs. rest of the country). Tabela I. Częstość występowania (%) niedowagi, masy ciała w normie, nadwagi i otyłości u dzieci i młodzieży w wieku 6-19 lat w zależności od płci i regionu zamieszkania (Polska Wschodnia vs. reszta kraju).

Gender *Płeć*	Nutritional status *Stan odżywienia*	Eastern Poland *Polska Wschodnia* % (N)	rest of the country *reszta kraju* % (N)	OR* (95%CI)	p
boys *chłopcy*	underweight *niedowaga*	11.2 (220)	9.7 (622)	1.17 (0.99-1.39)	0.053
	normal weight *masa* *ciała* *w normie*	72.5 (1425)	70.9 (4550)	1.08 (0.97-1.21)	0.168
	overweight *nadwaga*	13.0 (255)	14.8 (951)	0.86 (0.74-1.00)	**0.042**
	obesity *otyłość*	3.4 (66)	4.6 (297)	0.71 (0.54-0.95)	**0.016**
	overweight+obesity *nadwaga+otyłość*	16.3 (321)	19.4 (1248)	0.81(0.71-0.93)	**0.002**
girls *dziewczęta*	underweight *niedowaga*	15.2 (333)	13.3 (926)	1.16 (1.01-1.34)	**0.027**
	normal weight *masa* *ciała* *w normie*	71.6 (1573)	72.1 (5021)	0.97 (0.88-1.09)	0.640
	overweight *nadwaga*	10.7 (236)	11.9 (830)	0.89 (0.76-1.04)	0.133
	obesity *otyłość*	2.6 (56)	2.7 (189)	0.94 (0.68-1.28)	0.675
	overweight+obesity *nadwaga+otyłość*	13.3 (292)	14.6 (1019)	0.89 (0.78-1.03)	0.117
boys+girls *chłopcy +dziewczynki*	underweight *niedowaga*	13.3 (553)	11.6 (1548)	1.17 (1.05-1.30)	**0.003**
	normal weight *masa* *ciała* *w normie*	72.0 (2998)	71.5 (9571)	1.02 (0.95-1.11)	0.534
	overweight *nadwaga*	11.8 (491)	13.3 (1781)	0.87 (0.78-0.97)	**0.011**
	obesity *otyłość*	2.9 (122)	3.6 (486)	0.80 (0.65-0.98)	**0.031**
	overweight+obesity *nadwaga+otyłość*	14.7 (613)	16.9 (2267)	0.85 (0.77-0.93)	**<0.001**

* reference region – rest of the country N – number of pupils, OR – odds ratio, 95%CI – confidence interval, p – statistical significance N – liczba uczniów, OR – iloraz szans, 95%Cl - przedział ufności, p – istotność statystyczna

The analysis by gender indicated a higher risk of overweight (including obesity) among boys, as compared to girls, in both Eastern Poland (overweight − 13.0% vs. 10.7%: OR=1.24, 95% CI: 1.02-1.50, p=0.026; overweight + obesity − 16.3% vs. 13.3%: OR=1.27, 95% CI: 1.07-1.52, p=0.006) and the rest of the country (overweight − 14.8% vs. 11.9%: OR=1.29, 95% CI: 1.16 -1.42, p<0.001; obesity − 4.6% vs. 2.7%: OR=1.74, 95% CI: 1.44-2.10, p<0.001; overweight + obesity − 19.4% vs. 14.6%: OR=1.41, 95% CI: 1.28 -1.54, p<0.001). The girls (vs. boys) showed, in turn, a significantly higher rate of underweight both in the Eastern Poland region (15.2% vs. 11.2%: OR=1.42, 95% CI: 1.18-1.71, p<0.001) and the rest of the country (13.3% vs. 9.7 %: OR=1.43, 95% CI: 1.28-1.59, p<0.001). The mean standardized BMI in boys and girls from the Eastern Poland region for the entire age range was significantly lower in comparison with children and adolescents in the rest of the country, and the differences were: 0.09 z-score (p=0.001) in boys and 0.06 z-score (p=0.014) in girls (tab. II).

**Table II:** Differences in mean standardized BMI (z-score) of 6.5-18.5–year-old children and adolescents according to gender and region of residence. Tabela II. Różnice średniej standaryzowanej wskaźnika BMI (z-score) dla dzieci i młodzieży w wieku 6,5-18,5 lat według płci i regionu zamieszkania.

Variable *Zmienna*	Gender *Płeć*	Mean z-score (SD) *Średni z-score (SD)*		Mean difference *Różnica średnich*	Statistical significance *Istotność statystyczna*
		Eastern Poland *Polska Wschodnia*	Rest of the country *Reszta kraju*		
BMI	boys *chłopcy*	-0.07 (0.98)	0.02 (1.01)	0.09	**0.001**
	girls *dziewczęta*	-0.04 (1.01)	0.02 (1.00)	0.06	**0.014**
	boys+girls *chłopcy+* *dziewczęta*	-0.05 (0.99)	0.02 (1.01)	0.07	**<0.001**

## Discussion

The main result of the analysis is to demonstrate that there is a greater risk of overweight and obesity in children and adolescents living in regions of the country with higher GDP per capita. The differences observed were particularly significant in boys. The paper by Kułaga et al presenting preliminary results of the OLAF study, showed no significant differences in the prevalence of underweight and overweight and obesity depending on the region of residence, however, it showed a tendency consistent with the presented analysis − a larger prevalence of underweight in the Eastern Poland region, and overweight (including obesity) in the rest of the country [15]. Nevertheless, an analysis of the relationship between selected socioeconomic factors and thinness among Polish school-age children and adolescents shows that gender and the GDP region were important determinants of thinness [16]. The results of the analysis referring to the regional differences and identification of the areas of greater risk for overweight and obesity are consistent with the results of a nationwide survey conducted in 1995 among Polish children and adolescents [17]. The impact of the region of residence on the health of children and adolescents − and particularly on the nutritional status – is of interest to researchers from different countries [18, 19]. Regional differences of overweight (including obesity) frequency in children was reported between the former East and West Germany, with a significantly higher prevalence of overweight in Munich in comparison to Dresden (23.1% vs. 14.7%, p<0.0001) [20], while the tendency and the level of increase in the incidence of overweight and obesity after the reunification of the country were similar [21], which may be related to the improved economic situation and the adoption of the western lifestyle in the eastern part. The relationship between the socioeconomic status (SES) and health status of the paediatric population has been known for a long time [22]. It is believed that in developed countries, high BMI is associated with areas of a lower socioeconomic status, while the opposite is true in developing countries [23, 24]. British studies report that children from the group of lower income status were characterized by a significantly higher proportion of overweight and obesity in comparison to children from families with higher income (23.2% vs. 14.6%, respectively, p<0.001) [25]. In Australia low SES and middle SES were independent predictors of obesity in children and adolescents [26]. In the adult population of Eastern Europe, GDP per capita is positively and significantly associated with obesity (men: r=0.975, p<0.001, women: r=0.764, p <0.05) [27]. Egger et al. showed an initial close positive relationship between BMI and GDP per capita at low levels of GDP, followed by a levelling off at higher levels (no relationship at higher GDPs) [28]. A study by Wang et al regarding the incidence of overweight and underweight among children and adolescents aged 6-18 in four countries with different levels of economic development showed an increased incidence of overweight and decreased incidence of underweight in the U.S., China and Brazil − with a steadily rising GDP per capita, while in Russia, where a decline in GDP is occurring, a reduction in the prevalence of overweight and an increased prevalence of underweight is observed (only for girls) [29]. Noёl Cameron in his discussion of obesity among children in developing countries draws attention to the changes in these countries in the direction of rapid urbanization and acquisition of patterns related to nutrition and activity typical for developed countries, which inevitably leads to obesity and therefore increased health risks associated with the consequences of overweight in children and adolescents [30]. This is consistent with the conclusions of other authors, recommending the adjustment of national efforts in the field of food and nutrition to new realities, where the areas previously struggling with the problem of malnutrition are faced with the problem of overweight and obesity, which already affects children and adolescents in countries with different levels of economic development [24, 29]. Research confirmsthat not only the presence of overweight in childhood, but also the presence of childhood underweight and overweight in adulthood, is becoming an important health risk factor [31]. Therefore, making it possible to achieve and maintain normal body weight, both in children, adolescents and the adult population is becoming an important task of public health.

Our results also show the importance of the childhood overweight and obesity definition. We used the international definition, whereas analysis of social disparities, which applied to the OLAF study dataset childhood overweight definition based on national BMI references [32], shows the different pattern of GDP influence on the overweight and obesity rate across genders. This observation underlines the need for more research on the identification of body weight disturbances risk factors according to the different childhood overweight definitions.

## Conclusions

Identification of the diverse prevalence of overweight and underweight depending on the region, determined according to the economic status, becomes an opportunity to modify and adapt the strategy of implementing programs aimed at promoting healthy behaviours.
